# Validated HPTLC Method for Quantification of Luteolin and Apigenin in *Premna mucronata* Roxb., Verbenaceae

**DOI:** 10.1155/2015/682365

**Published:** 2015-09-03

**Authors:** Nayan G. Patel, Kalpana G. Patel, Kirti V. Patel, Tejal R. Gandhi

**Affiliations:** ^1^Department of Pharmacology and Toxicology, Faculty of Pharmacy, Dharmsinh Desai University, College Road, Nadiad, Gujarat 387001, India; ^2^Department of Quality Assurance, Anand Pharmacy College, Shri Ram Krishna Seva Mandal Campus, Near Town Hall, Anand, Gujarat 388001, India; ^3^Department of Pharmacology, Pharmacy Department, The MS University of Baroda, Vadodara, Gujarat 390002, India; ^4^Department of Pharmacology, Anand Pharmacy College, Shri Ram Krishna Seva Mandal Campus, Near Town Hall, Anand, Gujarat 388001, India

## Abstract

A simple, rapid, and precise high-performance thin-layer chromatographic method was developed for quantitative estimation of luteolin and apigenin in *Premna mucronata* Roxb., family Verbenaceae. Separation was performed on silica gel 60 F_254_ HPTLC plates using toluene : ethyl acetate : formic acid (6 : 4 : 0.3) as mobile phase for elution of markers from extract. The determination was carried out in fluorescence mode using densitometric absorbance-reflection mode at 366 nm for both luteolin and apigenin. The methanolic extract of *Premna mucronata *was found to contain 10.2 mg/g % luteolin and 0.165 mg/g % of apigenin. The method was validated in terms of linearity, LOD and LOQ, accuracy, precision, and specificity. The calibration curve was found to be linear between 200 and 1000 ng/band for luteolin and 50 and 250 ng/band for apigenin. For luteolin and apigenin, the limit of detection was found to be 42.6 ng/band and 7.97 ng/band while the limit of quantitation was found to be 129.08 ng/band and 24.155 ng/band, respectively. This developed validated method is capable of quantifying and resolving luteolin and apigenin and can be applicable for routine analysis of extract and plant as a whole.

## 1. Introduction

Quality control of medicinal plants is highly essential to ensure authenticity, stability, and consistency. Safety and efficacy studies along with the standardization of bioactive extract on the basis of active principle or major compound(s) as a quality assurance parameter shall open up unlimited possibilities for herbal medicine in pharmacotherapeutics [1–3]. Standardization can be carried out by obtaining a chemical fingerprint/profile in terms of one or more marker compounds (chemical or biomarker). Use of chromatography for standardization of plant products was introduced by the WHO and is accepted as a strategy for identification and evaluation of the quality of plant medicines [4–6]. HPTLC is becoming a routine analytical technique because of its advantages of low operating cost, high sample throughput, simplicity and speed, the need for minimum sample clean-up, reproducibility, accuracy, and reliability [7–10].

*Premna mucronata*, family Verbenaceae, commonly known as Arni is a large shrub or small tree of about 20 to 25 feet's height found in Northern India, the Gangetic plains, Uttar Pradesh, Bihar, and Bengal province of India. Besides, it is also found in hill area from Kumaon to Bhutan up to the height of 5000 feet from sea level [11]. The plant possesses antioxidant and hypocholesterolemics activities and is also known for improving digestion, acts as a blood purifier, cardiac stimulant, and expectorant, and is also useful in skin disorders [11–13]. It has been reported that the plant* Premna mucronata* contains flavonoids, luteolin, apigenin, clerodendrin, hispidulin, and pectolinarigenin. The plant also contains clerodin, clerosterol, D-mannitol, palmitic acid, ceryl alcohol, and cerotic acid. Within the complex mixture of phytoconstituents in this plant, luteolin and apigenin can be used as analytical markers for determination of its quality. Preliminary studies performed at our laboratory have established the efficacy of the plant* Premna mucronata* in cardioprotection against experimentally induced myocardial infarction in rats by isoprenaline and coronary artery ligation method [14–18]. Literature survey also has revealed that the flavonoid glycosides possess antioxidant properties and strong scavenging properties for superoxide radicals [19–22]. Moreover, luteolin, a flavonoid, is also capable of protecting the myocardium against IR injury, partly mediated through downregulation of NO production and its own antioxidant properties [16]. Hence, further work was focused on identification and quantification of flavonoids, luteolin and apigenin, in* Premna mucronata*. However, to the best of our knowledge, there is no report on thin-layer chromatographic method for quantitative analysis of luteolin and apigenin in methanolic extract prepared from whole plant* Premna mucronata*. Here, we have developed HPTLC method for quantitative estimation of luteolin and apigenin. The developed method was also validated in terms of system suitability, specificity, linearity, LOD, and LOQ according to ICH guidelines [23].

## 2. Materials and Methods

### 2.1. Equipment

Linomat 5 applicator (CAMAG, Switzerland), twin trough chamber (20 × 10 cm; CAMAG, Switzerland), TLC scanner IV (CAMAG, Switzerland), winCATS version 1.4.6 software (CAMAG, Switzerland), microsyringe (Linomat syringe 659.0014, Hamilton-Bonaduz Schweiz, CAMAG, Switzerland), UV chamber (CAMAG, Switzerland), and precoated silica gel 60 F_254_ aluminium plates (20 × 10 cm, 100 *μ*m thickness; Merck, Darmstadt, Germany) were used in the study.

### 2.2. Reference Compounds and Chemicals

Reference standard luteolin (L9283-10MG), apigenin (A3145-5MG), and NP reagent (2-aminoethyl diphenylborinate) (D9754-1G) were obtained from Sigma Aldrich Ltd. Analytical grade solvents were obtained from SD Fine Chemicals. Precoated silica gel 60 F_254_ HPTLC aluminium plates (10 × 10 cm, 0.2 mm thick) were obtained from E. Merck Ltd. (Mumbai, India).

### 2.3. Plant

*Premna mucronata* was obtained from commercial supplier of Anand. The plant was identified and authenticated by Dr. Geetha K A, Directorate of Medicinal and Aromatic Plants Research, Boriavi, Anand, Gujarat, India.

### 2.4. Preparation of Standard Luteolin Solution

A stock solution of luteolin was prepared by dissolving 5 mg of accurately weighed luteolin in methanol and making up the volume to 10 mL with methanol. Working solution of luteolin (200 *μ*g/mL) was prepared by appropriate dilutions of the stock solution with methanol.

### 2.5. Preparation of Standard Apigenin Solution

A stock solution of apigenin was prepared by dissolving 5 mg of accurately weighed apigenin in methanol and making up the volume to 10 mL with methanol. The stock solution was further diluted with methanol to give a standard solution of apigenin (50 *μ*g/mL).

### 2.6. Chromatographic Conditions

Chromatographic conditions are as follows:

 Stationary phase: precoated silica gel 60 F_254_ HPTLC aluminium plates (10 × 10 cm, 0.2 mm thick).
 Mobile phase: toluene : ethyl acetate : formic acid (6 : 4 : 0.3).
 Saturation time: 15 minutes.
 Wavelength: 366 nm.
 Lamp: mercury.

The HPTLC analysis was performed in an air conditioned room maintained at 22°C and 55% humidity using precoated silica gel 60 F_254_ aluminium backed plates (10 × 10 cm, 0.2 mm layer thickness, 5-6 *μ*m particle size; Merck, Darmstadt, Germany). 6 *μ*L and 5 *μ*L of the standard solutions of luteolin and apigenin were spotted using a Linomat 5 autosampler fitted with a 100 *μ*m Hamilton syringe (CAMAG, Muttenz, Switzerland) and operated with settings of a band length of 8 mm; distance between bands of 5 mm; distance from the plate edge of 10 mm; and distance from the bottom of the plate of 10 mm. The plates were developed to a distance of 80 mm using toluene : ethyl acetate : formic acid (6 : 4 : 0.3, v/v) mobile phase in CAMAG twin trough chamber presaturated with mobile phase. The developed plates were air dried and scanned with a CAMAG TLC Scanner 4 equipped with winCATS planar chromatography manager (version 1.4.6) software that was used for densitometry measurements, spectra recording, and data processing. The absorption/remission measurement mode was used at a scan speed of 20 mm/s. Zones of luteolin and apigenin were scanned from 200 to 400 nm to record their absorption and fluorescence spectra, respectively. Densitogram was recorded in fluorescence mode for both luteolin and apigenin.

### 2.7. Calibration Curve for Standard Luteolin

The standard solution of luteolin (200 to 1000 ng/band) was applied in triplicate on HPTLC plate. The plate was developed and scanned as per the chromatographic conditions mentioned above. The peak areas were recorded. Calibration curve of luteolin was prepared by plotting peak area versus concentration of luteolin applied.

### 2.8. Calibration Curve for Standard Apigenin

The standard solution of apigenin (50 to 250 ng/band) was applied in triplicate on HPTLC plate. The plate was developed and scanned as per the chromatographic conditions mentioned above. The peak areas were recorded. Calibration curve of apigenin was prepared by plotting peak area versus concentration of apigenin applied.

### 2.9. Preparation of Methanolic Extract of* Premna mucronata* (MEPM)

Accurately weighed 10 gm of powdered drug of whole plant was extracted for 15 min with methanol (4 × 25 mL) under reflux on water bath at 100°C. The methanolic extract was filtered through Whatman number 1 filter paper. The filtrates were combined, concentrated, and transferred to a 50 mL volumetric flask, and the volume was made up to 50 mL with methanol.

### 2.10. HPTLC Analysis of Plant Extract

Samples of methanolic extract of* Premna mucronata* were filtered through 0.45 *μ*m filter, and HPTLC was performed under the conditions optimized for the reference compound. The plates after development were dried in air and photographed at 254 nm. The plates were then derivatized by spraying with NP-PEG reagent followed by photographing the plates in visible and fluorescence mode and scanned at 366 nm for both luteolin and apigenin. The amount of luteolin and apigenin in plant extract was quantified using calibration curve plotted with luteolin and apigenin.

### 2.11. Validation of the Method

The method was validated according to International Conference on Harmonization guidelines.

Linearity was studied by applying different aliquots of standard stock solution in the ranges 200 to 1000 ng/band for luteolin and 50 to 250 ng/band for apigenin. The calibration curves were developed by plotting peak area versus concentrations. The areas of peaks were treated by least square linear regression analysis. The limit of detection (LOD) and limit of quantification (LOQ) were determined using the following equations:

(1) $$\begin{array}{c} \text{LOD}=\frac{3.3\times \text{Standard Deviation of the}\;\;y\text{-intercept}}{\text{Slope of the calibration curve}},\\ \text{LOQ}=\frac{10\times \text{Standard Deviation of the}\;\;y\text{-intercept}}{\text{Slope of the calibration curve}}. \end{array}$$

The intermediate precision of the method was studied by analyzing aliquots of standard in triplicate at 3 concentration levels for luteolin and apigenin on the same day for intraday precision. The study was also repeated on different days with freshly prepared samples in order to determine interday precision. The results were expressed as relative standard deviation (RSD). The specificity of the method was ascertained by determining the peak purity of the component by overlaying the fluorescence spectra of luteolin and apigenin in the sample extract with the spectra of reference standard luteolin and apigenin at the start, middle, and end positions of the bands. The accuracy of the method was determined by recovery studies at 3 levels in triplicate. For recovery studies, known amount of standard was added.

### 2.12. Statistical Analysis

The statistical analysis was performed using Microsoft Excel 2007.

## 3. Results and Discussion

**Figure 1:**
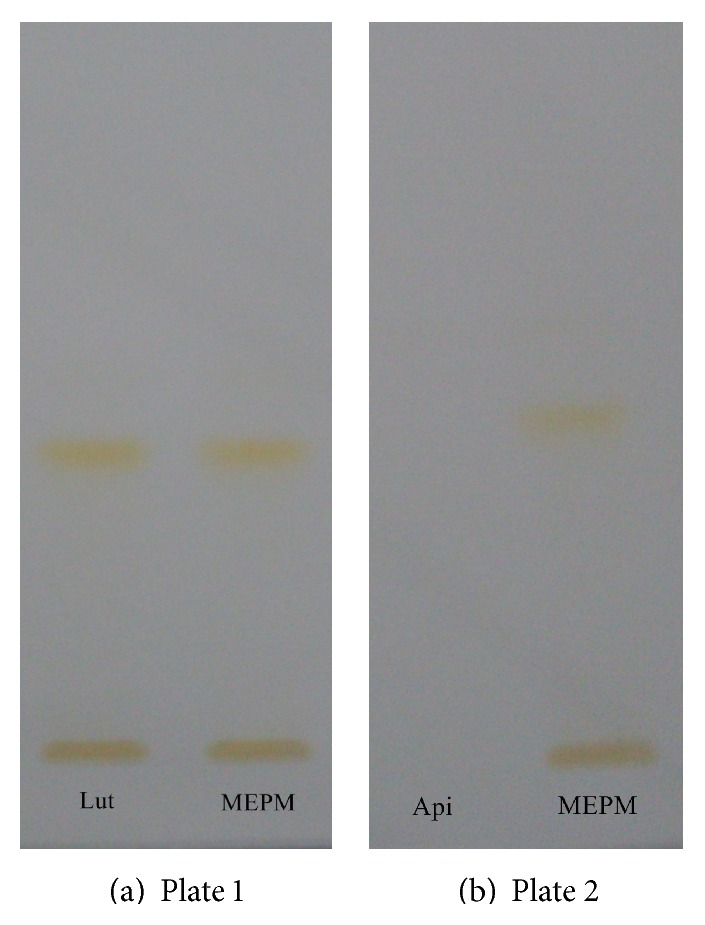
((a) and (b)) Visible mode after spray with NP-PEG reagent, Lut: luteolin standard, Api: apigenin standard, and MEPM: methanolic extract of* Premna mucronata*.

**Figure 2:**
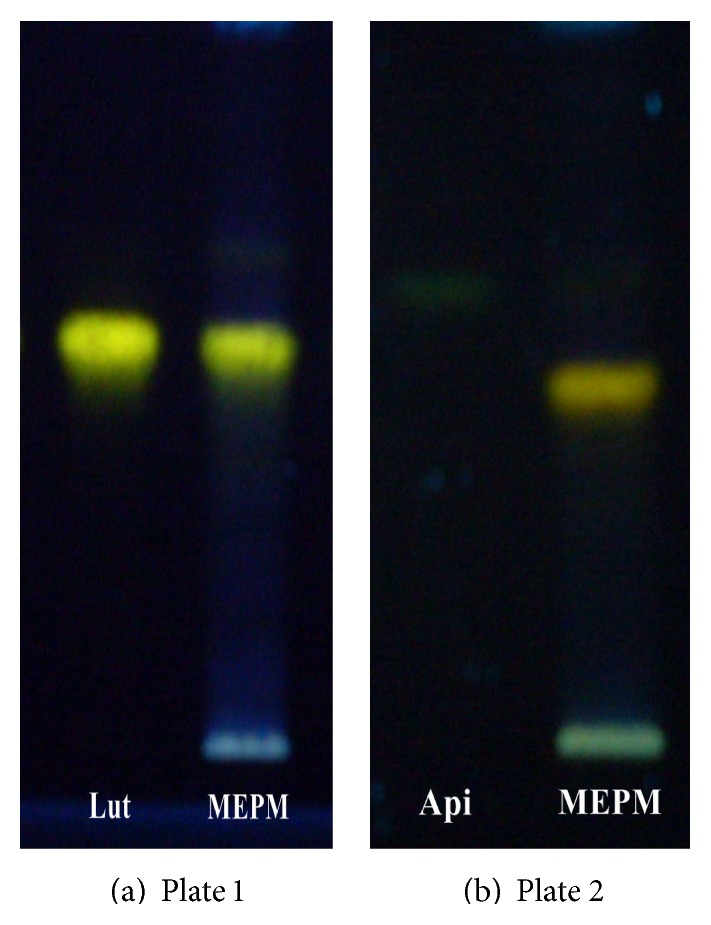
((a) and (b)) Fluorescence mode after spray with NP-PEG reagent, Lut: luteolin standard, Api: apigenin standard, and MEPM: methanolic extract of* Premna mucronata*.

**Figure 3:**
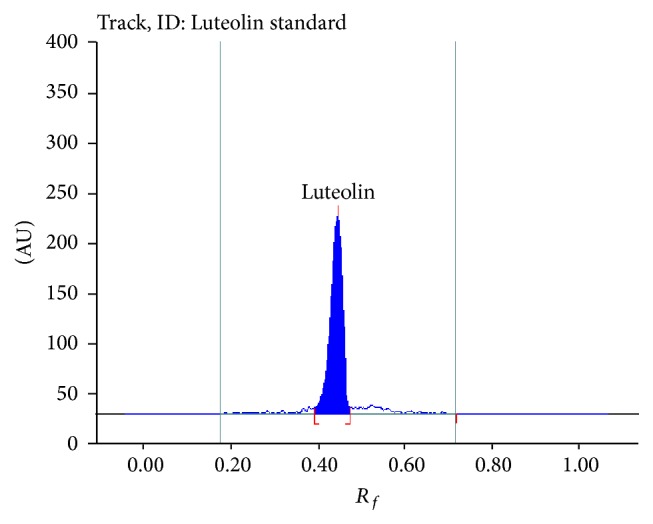
Chromatogram of luteolin standard.

**Figure 4:**
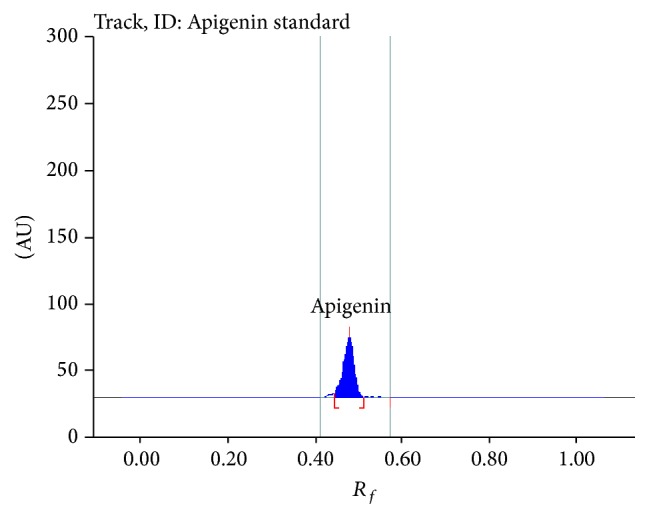
Chromatogram of apigenin standard.

**Figure 5:**
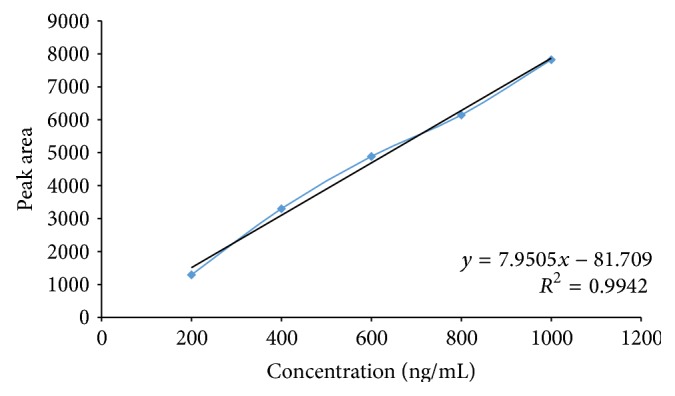
Calibration curve of luteolin standard.

**Figure 6:**
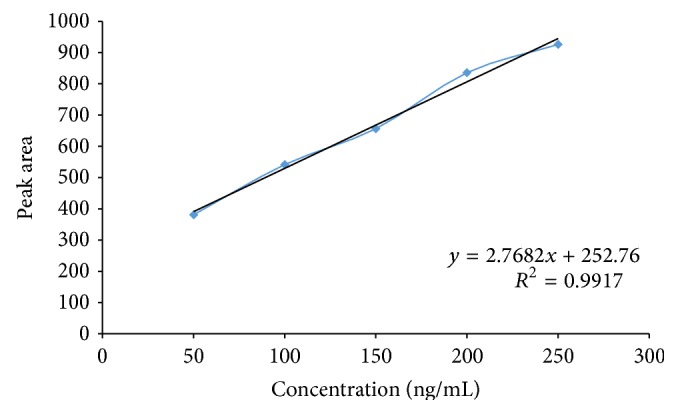
Calibration curve of apigenin standard.

Different proportions of toluene and ethyl acetate were tried as the mobile phase on silica gel HPTLC plates and a ratio of (6 : 4 v/v) gave good resolution. Well resolved symmetric band for luteolin and apigenin in extract was obtained under the optimized conditions using precoated HPTLC plates with 0.2 mm thickness, 5-6 mm particle size, and the mobile phase toluene : ethyl acetate : formic acid (6 : 4 : 0.3). Luteolin and apigenin on derivatization with NP-PEG reagent (natural products polyethylene glycol reagent) appeared orange in visible mode (Figures 1(a) and 1(b)) and gave bright yellow and parrot green fluorescence in fluorescence mode (Figures 2(a) and 2(b)) [24]. Standard luteolin (*R*_*f*_ 0.45) and standard apigenin (*R*_*f*_ 0.49) showed single peak in HPTLC chromatogram (Figures 3 and 4). Calibration curve of luteolin and apigenin was prepared by plotting concentration of luteolin versus area of the peak and concentration of apigenin versus area of the peak, respectively (Figures 5 and 6).

### 3.1. Determination of Marker in Extract

**Figure 7:**
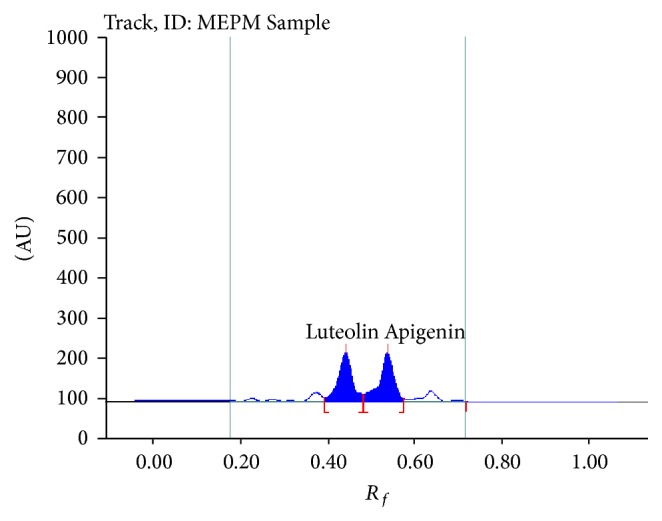
Chromatogram of methanolic extract of* Premna mucronata *Roxb. showing presence of luteolin (*R*_*f*_ 0.45) and apigenin (*R*_*f*_ 0.5).

The methanolic extract of* Premna mucronata* was found to contain 10.2 mg/g % luteolin and 0.165 mg/g % of apigenin. Furthermore, the methanolic extract of* Premna mucronata* shows peak in the chromatogram at same *R*_*f*_ value as luteolin (0.45) standard and apigenin (0.49) standard (Figure 7).

### 3.2. Linearity

Luteolin and apigenin showed good correlation coefficient of 0.9942 and 0.9917, respectively, when peak area of the resolved band was plotted against concentration, thus exhibiting good linearity between concentration and peak area.  Table 1 summarizes Beer's law limit, linear regression equation, and correlation coefficient for the method.

### 3.3. Precision

The proposed method was found to be precise as indicated by intermediate precision studies expressed as percent RSD (relative standard deviation) for intraday and interday variations as shown in Tables 2 and 3.

### 3.4. Accuracy

The proposed method when used for quantitation of marker after spiking with standard afforded recovery of luteolin and apigenin in the range of 96.67%–102.92% and 97.72%–99.29%, respectively, at three concentration levels as shown in Tables 4 and 5.

### 3.5. Limit of Detection and Limit of Quantification

For luteolin and apigenin, the limit of detection was found to be 42.6 ng/band and 7.97 ng/band while the limit of quantitation was found to be 129.08 ng/band and 24.155 ng/band, respectively.

### 3.6. Specificity

**Figure 8:**
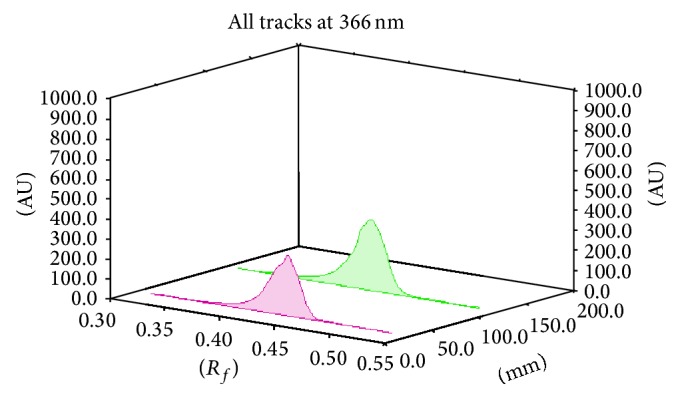
TLC densitograms for comparison of methanol extract of* Premna mucronata* Roxb. with reference standard, luteolin at 366 nm. Standard luteolin (shows peak at *R*_*f*_ 0.45), methanol extract (shows peak at *R*_*f*_ 0.45).

**Figure 9:**
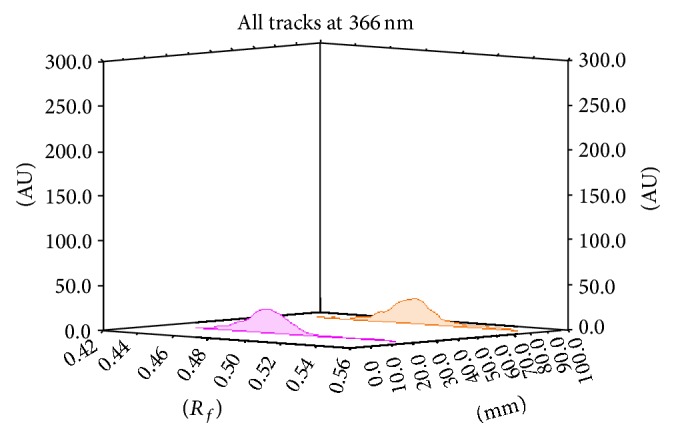
TLC densitograms for comparison of methanol extract of* Premna mucronata* Roxb. with reference standard, apigenin at 366 nm. Standard apigenin (shows peak at *R*_*f*_ 0.49), methanol extract (shows peak at *R*_*f*_ 0.49).

**Figure 10:**
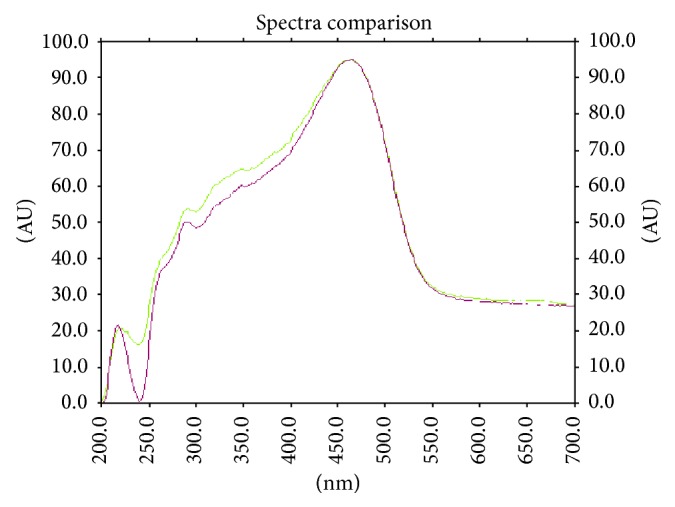
Overlain UV spectra at 254 nm for peak purity of luteolin.

**Figure 11:**
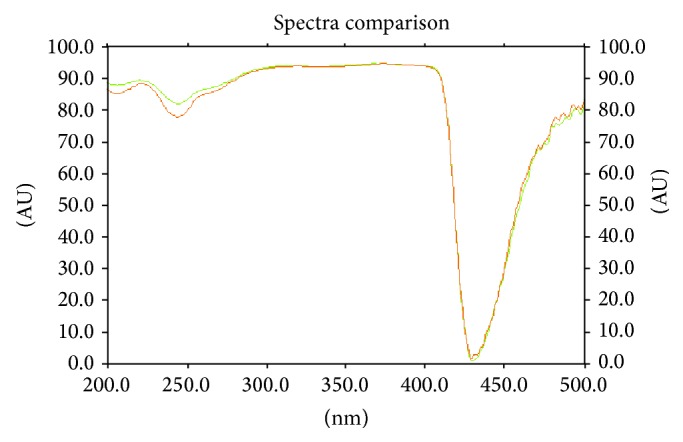
Overlain fluorescence spectra at 366 nm for peak purity of apigenin.

The identity of the bands in the sample extracts was confirmed by comparing *R*_*f*_, absorption spectra, and fluorescence spectra and by overlaying with those of their respective standard using CAMAG TLC Scanner 4. There were no interfering spots by the plant constituents at *R*_*f*_ values of the marker. The absorption spectra of standard marker luteolin (*R*_*f*_ 0.45) and apigenin (*R*_*f*_ 0.49) and the corresponding spot present in extract matched exactly, indicating no interference by the other plant constituents (Figures 8 and 9). The purity of the bands due to luteolin and apigenin in the sample extract was confirmed by overlaying the absorption and fluorescence spectra recorded at start, middle, and end position of the band in the sample tracks, respectively (Figures 10 and 11).

## 4. Conclusion

The proposed HPTLC method was found to be rapid, simple, and accurate for quantitative estimation of luteolin and apigenin. The recovery of luteolin and apigenin from whole part of* Premna mucronata* was found to be 96.67%–102.92% and 97.72%–99.29%, respectively, revealing accuracy of the developed method and hence can be applicable for routine analysis of extract and plant as a whole. Moreover, HPTLC profile by co-TLC can be used for fingerprinting thereby providing assurance of standardization and quality. And HPTLC has various advantages of low operating cost, high sample throughput, simplicity, and the need for minimum sample clean-up. In future, as the plant shows good cardioprotective activity, dosage form of the same can be formulated where fingerprinting and quantification of both the chemical markers will be used.

## Figures and Tables

**Table 1:** Method validation parameters for estimation of luteolin and apigenin.

Parameters	Luteolin	Apigenin
Wavelength, nm	366	366
Linearity range, ng/band	200–1000	50–250
Regression equation	*y* = 7.950*x* − 81.70	2.768*x* + 252.7
Correlation coefficient	0.9942	0.9917
Limit of detection, ng/band	42.6	7.97
Limit of quantification, ng/band	129.08	24.155
Specificity	Specific	Specific

**Table 2:** Intermediate precision studies for luteolin.

Concentration	Concentration^a^	Intraday^b^	Concentration^a^	Interday^b^
(ng/band)	(ng/band)	(% RSD)	(ng/band)	(% RSD)
400	402.3	1.08	403.69	1.32
600	604.3	0.57	606.71	0.65
800	798.8	0.38	799.20	0.43

^a^Mean of three determinations; ^b^% RSD—relative standard deviation.

**Table 3:** Intermediate precision studies for apigenin.

Concentration	Concentration^a^	Intraday^b^	Concentration^a^	Interday^b^
(ng/band)	(ng/band)	(% RSD)	(ng/band)	(% RSD)
100	101.43	0.67	101.3	0.60
150	150.3	0.43	150.3	0.41
200	201.1	0.27	201.15	0.18

^a^Mean of three determinations; ^b^% RSD—relative standard deviation.

**Table 4:** Recovery studies of luteolin.

Amount of luteolin in sample (ng)	Amount of standard luteolin added (ng)	Total amount of luteolin taken (ng)	Total amount of luteolin found (ng)	Percent of recovery^a^ (%)
454.89	200	654.89	633.13	96.67 ± 1.20
454.89	400	854.89	842.58	98.56 ± 0.69
454.89	600	1054.89	1085.66	102.92 ± 0.85

^a^Mean of three determinations ± % relative standard deviation.

**Table 5:** Recovery studies of apigenin.

Amount of apigenin in sample (ng)	Amount of standard apigenin added (ng)	Total amount of apigenin taken (ng)	Total amount of apigenin found (ng)	Percent of recovery^a^ (%)
131.57	100	231.57	229.91	99.29 ± 1.22
131.57	200	331.57	328.23	98.99 ± 1.21
131.57	300	431.57	421.77	97.72 ± 0.82

^a^Mean of three determinations ± % relative standard deviation.
